# Implementation evaluation of a Massachusetts pilot program designed to reduce overdose deaths among Black and Latino men returning from incarceration

**DOI:** 10.3389/fpubh.2026.1950283

**Published:** 2026-09-29

**Authors:** Laura McElherne, Andrés Hoyos-Céspedes, Shanyin Yang, Benjamin Goldberg, Sarah Jalbert, Ranjani Krishnan Paradise

**Affiliations:** 1 Institute for Community Health, Malden, MA, United States; 2 Cambridge Health Alliance, Cambridge, MA, United States; 3 Tufts University School of Medicine, Boston, MA, United States

**Keywords:** Black and Latino men, culturally responsive care, implementation evaluation, overdose prevention, reentry program, substance use disorders

## Abstract

**Introduction and context:**

In response to rising and disproportionate overdose deaths, a Massachusetts agency developed a program model (CARE) that incorporated evidence-based and evidence-informed practices to support recovery and overdose prevention for Black and Latino men returning to their communities from incarceration. Recruitment of Black and Latino men at risk of overdose was intended to begin in local jails prior to release. Key program components included culturally-responsive care, person-centered care, support for social determinants of health (SDoH), community building and social support, and facilitating access to evidence-based substance use disorder (SUD) treatment and harm reduction services. The evaluation used the RE-AIM framework to examine the program’s reach, adoption, and implementation among five community-based providers with varied sizes, populations of focus, prior experience with similar services, and institutional capacities.

**Evaluation methods:**

The evaluation used a participatory process to develop a theory of change for CARE and corresponding mixed-methods data collection procedures. Qualitative data were collected from semi-structured interviews and focus groups with 35 staff members, and interviews with 41 clients. Quantitative data sources included administrative program data on client characteristics, contacts, and service planning.

**Results:**

The evaluation found considerable inter-provider variation in recruitment of the focus population, with underrepresentation of Black men, and people with substance use histories that indicate highest overdose risk. All providers experienced challenges with recruitment and retention of staff. A minority of funded organizations established dedicated program space in alignment with the program design. Most providers experienced challenges with client recruitment in local jails. Providers successfully implemented culturally-responsive care and supporting SDoH needs. Implementation success was more mixed around community-building, the provision of person-centered care, and facilitating access to evidence-based substance use disorder treatment and harm reduction services.

**Discussion:**

The evaluation demonstrates that while the CARE program model is generally feasible, successful implementation of the core program elements was correlated with organizational size, longevity, experience with grant-funded program implementation and SUD service delivery, and physical infrastructure. Evaluation findings highlight lessons that can be used by funders and programs to effectively engage and serve populations at high risk of overdose, as well as future directions for further evaluation.

## Introduction

Drug overdose has been a significant cause of death in Massachusetts for over a decade, reflecting national trends (1). Although the opioid-related overdose death rate in Massachusetts declined slightly from 2015–2019, it increased by 3% from 2019 to 2020 and by 10% from 2020 to 2021, with 32.7 deaths per 100,000 people that year (2). Additionally, from 2019 to 2020, the opioid-related overdose death rate for non-Hispanic Black men rose from 32.6 to 58.8 per 100,000, surpassing that of non-Hispanic White men for the first time. The rate for Hispanic/Latino men was comparable to that of non-Hispanic Black men in 2020, and increased further to 65.4 per 100,000 in 2021 (3). These increases occurred in the early part of the COVID-19 pandemic, which created a range of circumstances that are linked to increased overdose risk, including social isolation, job loss, healthcare service disruptions, and mental health impacts (4–6). Massachusetts data also shows staggering disparities for people who have been incarcerated; analyses of public health data sets from 2011–2015 found that the opioid-related overdose death rate was 50–120 times higher for formerly incarcerated people than for the general population, with the risk of death being highest in the month following release (7, 8). These trends are consistent with findings from studies in other settings, which have also found a higher overdose death rate for formerly incarcerated people and a high risk of death in the weeks immediately following release (9, 10).

In response to the rising overdose death rates in 2020, a state agency developed the Culturally-Affirming Re-Entry (CARE) program^1^ to support recovery and overdose prevention for Black and Latino men with a history of substance use disorder (SUD) re-entering the community from a jail or prison. While Massachusetts, and particularly the Boston area, had many resources for harm reduction and SUD treatment, there were no programs at the time designed specifically to address both racial/ethnic and incarceration-related disparities. Further, while there were a wide range of reentry program models reported in the literature that incorporated pre- and post-release services, SUD interventions, support for social determinants of health, and/or case management (11–13), there were none to the state agency’s knowledge that focused exclusively and intentionally on Black and Latino men.

## Context

In July 2021, five community-based pilot sites (“providers”) in cities with high overdose death rates received funding to implement the program model over 5 years, with the first 3 years as a pilot period. Opioid-related overdose deaths in the funded organizations’ catchment areas ranged from 8 to 245 in 2020 (14). Provider characteristics at the time of the initial funding are summarized in Table 1. There were no strictures on how grantees could allocate their funding to staffing, facilities, and direct care. Budget allocations and staffing models therefore varied considerably despite identical initial funding, with some providers utilizing a small number of full-time frontline staff and others utilizing more part-time staff.

**Table 1:** Characteristics of the CARE providers.

Provider	Organizational characteristics	Populations of focus prior to the CARE program
Provider A	Established, mid-sized, community-based behavioral health center offering comprehensive inpatient and outpatient mental health and substance use disorder treatment and support services, as well as recovery-focused short-term and permanent supportive housing for more than three decades. Their direct care staff were all bilingual.	Spanish-speaking community members
Provider B	Small community-based organization providing mental health and substance use treatment through clinical counseling and coaching. The organization had been operating for about 10 years at the time of being funded, and had limited experience with SUD harm reduction or treatment approaches aside from clinical therapy and peer support groups. Eighty percent (80%) of client-facing staff were Black or Latino.	Fathers of color, particularly Black men
C1 and C2 (“Provider C”)	One primary grantee (C1) and one subcontractor (C2). Both organizations are large-scale Federally Qualified Health Centers (FQHC) providing comprehensive physical and behavioral health care, including evidence-based SUD treatment and harm reduction supports, in neighboring municipalities with diverse populations. Both organizations brought over five decades of experience.	C1 served a predominantly Hispanic/Latine immigrant community, and the majority of C2’s patients were Hispanic/Latine and 10% were Black. Both organizations primarily served a low-income patient population.
Provider D	Small and very young community-based organization that, at the time of receiving this funding, was initially focused on violence interruption, mentorship, and life skills development, and had limited experience with SUD harm reduction, treatment, or recovery support. This grant more than doubled their total operating revenue. All staff were Black or Latino.	Young men of color
Provider E	Established, mid-sized, neighborhood-focused nonprofit providing comprehensive family and social support, community advocacy, and health and disability services. The organization had experience with SUD recovery support and harm reduction programming. More than 80% of their staff were bilingual Spanish speakers.	Local Latine community

## Intervention design

### Eligibility criteria

The intended participant population was individuals ages 18 or older who (1) identified as male, (2) identified as Black and/or Latino, (3) had a history of substance misuse indicating overdose risk, and (4) were re-entering from or had previously been incarcerated in a Massachusetts House of Correction (HOC) or Department of Correction (DOC) facility. Exclusion criteria included inability to consent to participate in services, and immediate threats to the physical safety of staff and other participants. There were no exclusion criteria related to current substance use or criminal history. The program model envisioned providers engaging participants and initiating reentry planning approximately 6 months pre-release, with reentry service provision beginning at the providers’ community-based locations immediately upon release.

### Key program components

The CARE program model was designed to incorporate several evidence-based or evidence-informed practices that support recovery and re-entry for the focus population, with specific implementation guidelines developed by the state agency (Table 2).

**Table 2:** CARE program components.

Key component	Justification	Description of intended implementation for CARE
Culturally- responsive care	Culturally-responsive care facilitates participant satisfaction and trust and can be effective at preventing or reducing substance use when applied to specific interventions (29, 35). Socially concordant staffing also supports rapport-building with re-entering clients and is associated with reduced substance use, improved satisfaction with SUD care, and increased retention in SUD treatment (36, 37).	• Engagement, service provision, and community-building activities are designed to center and affirm the intended recipients’ distinct cultural backgrounds.
		• Staff reflect the intended participant population; e.g., self-identify as Black, Latino, male; have lived or living experience with addiction or recovery; have criminal-legal system history; and/or speak Spanish or other languages spoken by clients.
Person- centered care	Associated with increased utilization of treatment and support services at SUD treatment clinics and with indicators of reduced substance use (38, 39)	• Providers work with people at all stages of recovery; sobriety is not required for engagement with the program.
		• Participants and staff co-develop an Individualized Service Plan (ISP) to articulate the participant’s own goals and strategies for meeting those goals, based on their unique needs and history.
		• ISPs are revisited periodically to monitor and celebrate progress toward goals, and to adapt goals as priorities change.
		• Engagement, service provision, and community-building activities focus on participant-identified goals and priorities.
		• Participant feedback on programmatic activities is sought and acted upon.
		• Staff connect with participants frequently, with a goal of weekly engagement for the first 6 months of enrollment.
		• Staff conduct outreach to participants whose engagement declines.
Addressing social determinants of health (SDOH)	Higher social vulnerability and SDOH needs are associated with increased substance use prevalence, overdose risk, and overdose mortality (40–42)	• Programs provide comprehensive care coordination, including supporting referrals and linkages to care for any social services related to the client’s goals (in addition to substance use disorder, mental health, and medical services).
		• Services may be provided in-house at the provider’s organization or externally by community partner agencies.
Community- building and social support	Associated with greater readiness to change, improved SUD treatment outcomes, reduced substance use, and reduced overdose risk (42–46)	• Providers offer group-based and community-building activities, such as peer support groups and culturally responsive social events.
		• Providers maintain a dedicated physical space where participants can gather to receive services and participate in social activities, with a goal of fostering brotherhood and a sense of community.
Facilitating access to evidence based treatment and harm reduction services	Medications for opioid use disorder are associated with reduced all-cause and overdose-related mortality, opioid use, injection drug, and criminality (47); Naloxone distribution reduces opioid-related mortality (48); Syringe exchange services are associated with reduced risk behaviors and HIV infection (49, 50); and fentanyl test strips are associated with changes in drug use behavior that can reduce overdose risk (51, 52).	• Programs provide access to comprehensive, evidence-based options for treating substance use disorders, either in-house or through warm referrals to community partner agencies.
		• Programs provide harm reduction education and resources to support safer use, with connections to community-based services as appropriate.

## Evaluation methods

### Evaluation design

An external evaluation team conducted a mixed-methods implementation evaluation of the three-year pilot period (July 2021–June 2024) to understand the extent to which providers were able to implement the CARE model as designed, how they operationalized the program components, and implementation barriers and facilitators. Grounded in Equitable Evaluation Framework (15) principles, the evaluation aimed to (1) create opportunities for provider participation, (2) center staff and client lived experiences, (3) prioritize feasibility and minimize data collection burden, and (4) facilitate providers’ access to information.

In 2021, the evaluators facilitated a participatory process with provider staff and state stakeholders to visualize CARE’s theory of change in a logic model. This aligned stakeholders around CARE’s activities and intended outcomes to frame the evaluation. Next, evaluators identified implementation indicators, and worked with the state and providers to develop data collection procedures. In February 2022, the evaluation team initiated monthly meetings with each provider to provide database training, conduct quality assurance, co-design activities, collect qualitative data on successes and challenges, and interpret trends. Evaluators presented findings to the state agency and providers annually to co-interpret findings, discuss emerging areas of inquiry, and facilitate use of data for program improvement.

The state agency’s Institutional Review Board reviewed all study protocols and determined they were not human subjects research.

### Data collection and analysis

#### Program data

Providers documented their work in two systems: (1) A state database collected standardized intake, enrollment, and disenrollment forms including demographic and substance use items. Separate forms were used for incarcerated and community-based clients to track points of engagement. (2) Providers used an internal case management database to document client contacts, individual service plan goals, and notes on incarceration history. Data were linked and de-identified prior to analysis using 42 CFR Part 2-compliant protocols, and cleaned and analyzed using SAS 9.4. This included removing duplicates, standardizing variable names and values, and addressing missing data and correcting inconsistencies through consultation with program staff. Program data were analyzed using descriptive statistics to summarize the basic features of the sample and trends over time. Results were checked by other members of the study team, and member checked by program staff. The quantitative data covers July 2021–December 2023.

#### Staff and client interviews

In June–July 2022, the evaluation team conducted interviews with 24 CARE clients and 25 staff across providers. Frontline staff were offered the option to participate in individual interviews or focus groups (FGs); seven of the 25 chose to participate in three FGs in the first year. In March–May 2024, the team interviewed 17 clients and 19 individual staff, of which four staff participated in two FGs. Nine staff who maintained employment with the program through the study period participated in both rounds of data collection. Interviews/FGs were facilitated in English or Spanish using semi-structured guides, and client interview questions were pilot tested with provider staff.

Staff interviews and FGs were conducted by Zoom videoconference. All management staff who regularly participated in the monthly check-ins and all frontline staff were invited to participate.

Client interviews were conducted in person at providers’ sites using a convenience sample. Eligibility criteria included enrollment into CARE at least 1 month prior to the interview and the ability to converse in English or Spanish. Participants received a $50 Visa gift card. Interviews/FGs typically lasted 30–60 min. Interviews/FGs were audio recorded and professionally transcribed, and Spanish transcripts were professionally translated to English. Bilingual team members who facilitated the Spanish interviews checked the accuracy of transcripts and their translation to English.

Qualitative results were validated through a team-based analysis process. Transcripts were coded using Dedoose software according to domains developed deductively from the program logic model and from interview guides. Team members independently coded several representative transcripts, then met to compare applications and resolve discrepancies. Coded excerpts were analyzed to identify major themes around the program’s reach, implementation, adoption, and effectiveness; areas of strength and challenge; and factors influencing implementation. Themes were compared across providers to identify commonalities and differences. The team discussed and built consensus around emerging themes. Findings were further validated through member checking with program staff.

#### Evaluation synthesis

The evaluation team used the RE-AIM framework (16) to organize and synthesize information across data sources. RE-AIM includes five key outcomes: Reach, Effectiveness, Adoption, Implementation and Maintenance. This evaluation focused on Reach, Adoption, and Implementation. Maintenance was not included, as the study ended partway through the program funding period before sustainability could be assessed. Effectiveness was not included, because although staff and participants attributed positive impacts to CARE, the study was not designed to evaluate outcomes or establish causality.

### Limitations

Quantitative analysis was limited to participants entered into both databases with matching identifiers. Data entry practices were inconsistent between individual staff and providers and may underestimate provider effort. Contact data entry for incarcerated clients was inconsistent; our analysis of contacts thus only includes those that occurred post-release. Provider A was unable to provide complete contacts data for the evaluation data period, and is not included in the contacts analysis. Incarceration history was also not analyzed due to substantial missing data. For the analyses that were possible based on the available data, the member checking process with program staff validated findings against staff’s own knowledge of their service delivery. As such, the evaluation team determined the findings included here to be of sufficient quality for reporting and interpretation. Additionally, the use of a convenience sample for client interviews introduced potential selection bias: clients with more logistical barriers or negative experiences may be underrepresented. Findings should not be assumed to represent the full range of participant experience with CARE. Finally, this study was context-specific, and findings may not be generalizable, and the participatory design may have introduced social desirability bias.

## Results

### Reach

#### Total enrollments

Table 3 shows enrollment figures and client characteristics. Over the 2.5-year evaluation period, providers enrolled 680 unique clients. During this period, 29,122 pre-trial and sentenced people were released from HOCs in the providers’ counties, of which an estimated 4,409–6,532 were Black or Latino men with SUD.

**Table 3:** Demographic characteristics and self-reported substance use history at enrollment (*N* = 679).

Category	Provider A		Provider B		Provider C		Provider D		Provider E		All providers	
	*N*	%	*N*	%	*N*	%	*N*	%	*N*	%	*N*	%
Total enrollments	87	100	106	100	99	100	71	100	316	100	679	100
Initial enrollment pre-release	17	19.5	35	33.0	10	10.1	34	47.9	98	31.0	194	28.6
Initial enrollment post-release	70	80.5	71	67.0	89	89.9	37	52.1	218	69.0	485	71.4
Active caseload												
Peak caseload	61		59		74		34		97		283	
Time to reach peak (weeks)	125.0		66.9		128.7		81.1		63.9		125.0	
Mean caseload, year 3 (std. dev)	50.9 (7.3)		44.2 (5.2)		52.6 (10.1)		26.8 (3.2)		68.8 (6.5)		243.4 (14.6)	
Race/ethnicity												
Black, non-Hispanic/Latino	6	6.9	77	73.3	10	10.2	22	31.4	71	22.5	186	27.5
Black and Hispanic/Latino	9	10.3	6	5.7	7	7.1	10	14.3	5	1.6	37	5.5
Hispanic/Latino, not Black	66	75.9	19	18.1	76	77.6	36	51.4	236	74.7	433	64.1
Other race/ethnicity	6	6.9	3	2.9	5	5.1	2	2.9	4	1.3	20	3.0
Missing	0		1		1		1		0		3	
Age												
18–25	4	4.6	14	13.2	0	0.0	19	28.4	19	6.0	56	8.4
26–35	20	23.0	55	51.9	34	34.7	23	34.3	76	24.1	208	31.1
36–45	33	37.9	25	23.6	34	34.7	13	19.4	99	31.3	204	30.5
46–55	21	24.1	10	9.4	18	18.4	11	16.4	73	23.1	133	19.9
56–65	8	9.2	2	1.9	4	4.1	1	1.5	49	15.5	64	9.6
65 or more	1	1.1	0	0.0	3	3.1	0	0.0	0	0.0	4	0.6
Missing	0		1		1		4		0		6	
Has children	64	73.6	84	80.0	75	75.8	44	63.8	248	78.7	515	76.0
Missing	0		0		0		0		1		1	
Highest educational attainment												
No formal education	2	2.4	1	1.1	9	9.1	4	5.9	5	1.6	21	3.2
Some schooling	15	18.3	6	6.3	19	19.2	18	26.5	47	15.3	105	16.0
Some high school	23	28.0	19	20.0	29	29.3	40	58.8	104	33.9	215	32.8
High school diploma/GED	36	43.9	51	53.7	34	34.3	0	0.0	124	40.4	245	37.4
Associates degree	0	0.1	1	1.1	1	1.0	5	7.4	3	1.0	10	1.5
Some college	5	6.1	15	15.8	7	7.1	1	1.5	19	6.2	47	7.2
College degree or higher	1	1.2	0	0.0	0	0.0	0	0.0	4	1.3	5	0.8
Other credentials	0	0.0	2	2.1	0	0.0	4	5.9	1	0.3	7	1.1
Missing	5		11		0		3		9		28	
Housing status*												
Group home or treatment	26	36.6	2	2.5	1	1.1	1	2.0	45	18.1	75	13.9
House/Apartment	23	32.4	52	64.2	47	53.4	33	64.7	92	36.9	247	45.7
Institution	3	4.2	5	6.2	0	0.0	1	2.0	3	1.2	12	2.2
Room/Boarding or sober house	10	14.1	15	18.5	20	22.7	12	23.5	55	22.1	112	20.7
Shelter/Mission	3	4.2	3	3.7	9	10.2	2	3.9	14	5.6	31	5.7
Streets	6	8.5	4	4.9	11	12.5	2	3.9	40	16.1	63	11.7
Missing	0		0		1		0		0		1	
Employment status*												
Not in labor force: disabled	1	1.4	1	1.2	9	10.2	2	3.9	46	18.5	59	11.0
Not in labor force: homemaker	0	0.0	0	0.0	0	0.0	1	2.0	0	0.0	1	0.2
Not in labor force: other	0	0.0	3	3.7	3	3.4	6	11.8	15	6.0	27	5.1
Not in labor force: student	0	0.0	0	0.0	0	0.0	0	0.0	1	0.4	1	0.2
Unemployed: looking	43	60.6	43	53.1	51	58.0	23	45.1	128	51.6	288	53.9
Unemployed: not looking	22	31.0	4	4.9	14	15.9	5	9.8	39	15.7	84	15.7
Working full time	2	2.8	17	21.0	6	6.8	3	5.9	10	4.0	38	7.1
Working part time	3	4.2	6	7.4	4	4.5	9	17.6	7	2.8	29	5.4
Other	0	0.0	2	2.5	1	1.1	2	3.9	2	0.8	7	1.3
Missing	0		0		1		0		1		2	
Lifetime substance misuse												
Opioids	57	65.5	19	17.9	75	75.8	29	42.0	203	64.2	383	56.4
Stimulants	60	69.0	21	19.8	81	81.8	44	63.8	231	73.1	437	64.4
Sedatives	7	8.1	4	3.8	20	20.2	10	14.5	58	18.4	99	14.6
Alcohol	51	58.6	76	71.7	72	72.7	66	95.7	257	81.3	522	76.9
Cannabis	56	64.4	88	83.0	85	85.9	65	94.2	256	81.0	550	81.0
Nicotine/tobacco	11	12.6	40	37.7	70	70.7	50	72.5	230	72.8	401	59.1
Missing	0		0		0		2		0		2	
Experienced overdose												
Lifetime	38	43.7	4	3.8	40	40.4	18	26.1	119	37.7	219	32.3
Past year	12	13.8	0	0.0	15	15.2	7	9.9	36	11.4	70	10.3
Estimated percentage reach among eligible release population in relevant county, Jul 2021–Dec 2023^†^												
Numerator	68	2.4, 3.7	30	1.1, 1.6	82	7.3, 9.2	47	5.4, 9.2	266	15.8, 22.7	493	7.5, 11.2
Denominator	1,843; 2,846		1,843; 2,846		887; 1,122		507; 877		1,169; 1,686		4,408; 6,532	

Percentages are out of total responses for the variable in question, excluding missing responses. One client with significant missing data is not included. *Housing and employment data were only tabulated for clients who had a community (post-release) enrollment (*N* = 538 in total; *N* = 71 for Provider A; *N* = 81 for Provider B; *N* = 89 for Provider C; *N* = 51 for Provider D; *N* = 249 for Provider E). For clients with multiple community enrollments, demographics were taken from the first enrollment unless otherwise noted. ^†^Numerator includes only CARE participants who met eligibility criteria around race/ethnicity and substance misuse history, which includes lifetime misuse of opioids, stimulants, or sedatives. Denominator includes potentially eligible population, estimated using selected community-release events from the combined sentenced and pre-trial population in four HOC catchments from July 2021 through December 2023 (53). Monthly county demographic data were used to estimate Black and/or Latino male release events, with lower and upper estimates reflecting possible overlap between the two groups. We then applied a 37% prevalence of drug use disorder among male prisoners in the United States (54), using 26 and 48% for sensitivity analyses. Ranges are reported as [lower bound, upper bound].

There was considerable variation among providers in total enrollments, ranging from 71 clients at Provider D to 317 at Provider E. Providers B and E reached their caseload peaks soonest, at 66.9 and 63.9 weeks, respectively. Provider E had the highest average caseload size over the last year of the evaluation period (68.8). Providers A and B retained moderate end-of-period mean caseloads, though as a proportion of the estimated eligible population in their county, their overall reach was the lowest. Provider D had the lowest peak caseload (*n* = 34), and lowest end-of-period mean caseload (26.8), although their proportional reach was comparable to that for Provider C. Only Provider D’s reach exceeded 10% of the estimated eligible population in their county.

#### Client characteristics

Most clients were first engaged post-release in the community (71.5%). Overall, 64.1% identified as non-Black Hispanic/Latino, 27.5% as non-Hispanic/Latino Black, and 5.5% both Black and Hispanic/Latino. Most (61.2%) were ages 26–45 at enrollment, and 76.3% were fathers. Educational attainment was low; only 10% had education beyond high school. Over half (53.9%) were unemployed and seeking work. Alcohol (77.1%), cannabis (81.2%), stimulants (64.5%), tobacco/nicotine (59.2%), and opioids (56.6%) were the most common substances of self-reported lifetime misuse. Just under one-third (32.3%) had experienced a lifetime overdose.

Client characteristics varied substantially among providers. Three (A, C, and E) recruited significantly more Latino men than Black men, while only Provider B served a majority of Black men. This reflected local demographics and providers’ historical focus populations. Additionally, Providers B and D recruited the lowest proportions of clients with a history of opioid misuse (17.9 and 42.0%, respectively) and history of overdose (3.8 and 26.1%, respectively). This stemmed from recruitment practices: during the first 2 years, Provider B focused on fathers with a history of alcohol use disorder, while Provider D did not incorporate substance use history into eligibility screening. In year two, the funder instructed both programs to recruit higher-risk participants.

#### Engagement while incarcerated

CARE envisioned providers building relationships with incarcerated people pre-release to be positioned to address their needs during the vulnerable post-release transition. Pre-release enrollment varied, from 10.1% of new clients at Provider C to 47.9% at Provider D.

Effective pre-release recruitment depended on providers’ ability to build productive working relationships with HOC partners. Providers D and E established the most consistent presence at their local jails, sending staff twice weekly for one-on-one client meetings and holding monthly presentations on their services. Provider D also hosted quarterly HOC stakeholder meetings, and Provider E ran a weekly HOC support group. Word-of-mouth in the HOCs further contributed to recruitment for both.

Providers A and B held weekly HOC support groups, but were unable to advertise CARE services facility-wide, and relied on HOC staff to select weekly attendees. Provider C was not able to acquire the required certification for physical jail access and instead introduced themselves via Zoom to participants referred by their local HOC’s Medication-Assisted Treatment program coordinator.

### Adoption

With RE-AIM, adoption is typically evaluated by examining the number and characteristics of settings that are willing or unwilling to initiate a program. However, because the CARE evaluation only included those who initiated the program, the evaluation team assessed adoption by examining how well providers established the infrastructure required to effectively implement the program, including physical space and staffing.

#### Physical space

CARE providers (see Table 1 for additional details on organizational characteristics at the time of funding) were expected to provide a dedicated drop-in communal program space to maintain cultural specificity and support community-building. However, only providers C1 and E designated dedicated space for CARE. Provider A’s services were delivered on its residential SUD treatment and sober housing campus where nearly all clients lived and there was no CARE-specific communal space. This co-location limited participants’ ability to meaningfully control their engagement with the program, and hindered the provider’s ability to provide low-barrier services across all stages of use and recovery. Providers B and D offered some off-site CARE-specific group programming, and Provider D held many CARE-specific groups and events in their offices. Though both providers’ office space was open for CARE drop-ins, the space was shared with other initiatives. Provider C2 lacked private office space and dedicated communal space, relying on health center conference rooms for groups.

#### Staffing

Staffing models varied across providers, but all organizations maintained a program director for leadership, oversight, and staff supervision. Several also hired a program manager for overall program coordination and additional supervision. Most client-facing work was conducted by one or more care coordinators. Providers experienced challenges with hiring and turnover across roles. In interviews, staff attributed challenges to (1) funding constraints that resulted in poor wages for staff and limited pathways for advancement, (2) staff burnout due to vicarious trauma exposure, and (3) difficulty finding applicants who brought relevant skills as well as mission- and values-orientation. As one staff person said, “It’s a heavy job, right? It’s heavy work that we do…and sometimes for the work that we do, like we should be rewarded a little bit more” (Provider D). Despite challenges, by the end of the pilot period all providers had in place a program director and at least two care coordinators.

Most providers prioritized hiring people with lived experience of SUD and/or incarceration. This facilitated trust and engagement for clients, even as it presented challenges. Staff with incarceration histories were prohibited from entering some HOC facilities, preventing their participation in pre-release recruitment and engagement. Additionally, some staff with less formal education and work experience struggled with some required administrative tasks.

Providers D and E also prioritized hiring staff with experience working in their local HOCs, and attributed their successful HOC recruitment to those staff’s experience and relationships.

### Implementation

#### Culturally-responsive care

Cultural responsiveness was central to the CARE approach, and was operationalized in three ways. First, providers established staff teams that reflected the culture of the intended client population to help build rapport and trust. In addition to gender and racial/ethnic concordance, staff understood culture to include living in similar neighborhoods and having experience with addiction and/or incarceration. Almost all staff shared affinity with clients in one or more of these ways.

While they had no strong preferences about staff gender or age, participants affirmed the importance of racial/ethnic concordance and lived experience. Shared backgrounds and experiences helped them relate to staff, making it easier to relax, communicate openly, trust staff’s advice, and gain inspiration, as one client described:

“Education, it only takes you so far…[when] you have not been an addict, you have not used, and you have not been sick from the drugs…you do not know what you are talking about because you have not been through it. And, personally, I will not listen to it or to a person who has not been through it…also with incarceration, if you have not been through it, you would not understand…here, people do…you have to wear the suit in order to understand it, and most everybody who works here is either Latino or Black, so people you grew up around. And, like I said, been through the same experiences, so you have that understanding…you see the positiveness of them, of them getting out from wherever they were at, coming out of jail or overcoming addiction, and you see that, and that makes you want to do the same thing. So, that helps a lot.” (Client interview, English, Provider D).

Next, providers worked to minimize language and communication barriers. They adapted to clients’ self-expression, hired bilingual staff, and translated materials into Spanish. Staff explained that speaking Spanish helps Latino clients feel comfortable and “drop their guard.” Providers also highlighted the importance of attending to communication nuances, being open rather than reinforcing specific cultural norms, and avoiding assumptions about clients. One staff elaborated on the impact of allowing clients to maintain their natural communication styles:

“I think it’s to meet a person where they are at without [them having to] put on different—filters…I think different kinds of jobs, at work, you have to speak or present in a different way. Or talk not to sound a little too this or too that. And [without that] you just feel that freedom.” (Staff interview, Provider A).

Finally, providers spoke about “respecting the differences,” recognizing the diversity of customs and cultures within and across racial/ethnic categories. They prioritized understanding each client’s specific background and finding ways to honor and appreciate this, such as offering culturally-specific foods, playing music that clients like, organizing activities that are popular in clients’ cultures, and learning about important client traditions.

#### Person-centered care and addressing SDOH

Providers tailored services to participants’ unique needs by developing Individualized Service Plans (ISPs). Overall, 69% of participants had an ISP documented, ranging from 31 and 32% at Providers B and D to 88% at Provider C (Table 4). Clients with ISPs averaged about three documented goals (from 1.3 at Provider D to 3.8 at Provider C).

**Table 4:** ISP completions, goals per client, and goal topics by provider.

Category	Provider A		Provider B		Provider C		Provider D		Provider E		All	
	*N*	%	*N*	%	*N*	%	*N*	%	*N*	%	*N*	%
Unique clients	87		106		99		71		316		679	
Clients with an ISP	70	80.5	33	31.1	87	87.9	23	32.4	256	81.0	469	69.1
Mean goals per client (std. dev)	2.4	(1.0)	3.2	(1.6)	3.8	(1.8)	1.3	(0.6)	3.1	(1.7)	3.0	(1.7)
Goal topic												
Basic needs	17	24.3	0	0	41	47.1	0	0	196	76.6	254	54.4
Housing	24	34.3	20	60.6	41	47.1	2	8.7	142	55.5	229	48.8
Education	10	14.3	5	15.2	7	8.0	7	30.4	143	55.9	172	36.7
Employment	20	28.6	15	45.5	36	41.4	8	34.8	73	28.5	152	32.4
IDs and documentation	18	25.7	21	63.6	28	32.2	0	0	57	22.3	124	26.4
Substance use recovery	23	32.9	1	3.0	41	47.1	9	39.1	31	12.1	105	22.4
Transportation	5	7.1	1	3.0	46	52.9	0	0	27	10.5	79	16.8
Mental health	13	18.6	4	12.1	16	18.4	0	0	23	9.	56	11.9
Physical health	0	0	6	18.2	33	37.9	0	0	14	5.5	53	11.3
Benefits and entitlements	10	14.3	3	9.1	20	23.0	0	0	13	5.1	46	9.8
Legal	20	28.6	8	24.2	2	2.3	0	0	1	0.4	31	6.6
Family	1	1.4	8	24.2	2	2.3	0	0	4	1.6	15	3.2
Community/social	0	0	1	3.0	2	2.3	4	17.4	8	3.1	15	3.2
Self-improvement	0	0	7	21.2	0	0	0	0	3	1.2	10	2.1
Spiritual	0	0	2	6.1	0	0	0	0	1	0.4	3	0.6

Clients identified goals across a range of categories. Across providers, the top five goal areas, each named by at least one quarter of clients, related to SDOHs: basic needs (54%), housing (49%), education (37%), employment (32%), and IDs/documentation (26%).

Interview data gave context on how providers supported participants with these goals. Material support for basic needs included food, clothing, toiletries, transportation, and cell phones, especially in the period immediately after release. Providers helped clients secure shelter and sober home beds and assisted with permanent housing applications. To address employment barriers related to criminal records, providers offered referrals to workforce development programs and built relationships with local employers and training programs. Finally, providers helped clients obtain identification documents by providing logistical and financial support with fines, fees, and back-due child support that blocked driver’s license acquisition.

Participants reported progress they attributed to the program in achieving and maintaining sobriety, finding housing and employment, and strengthening their relationships with their children and families. For example, one client said:

“Well, I got my family back in my life, and I’ve been sober. I accomplished a lot of goals…I did the recovery coach training…I got a certificate that I could work in courthouses and in hospitals. And culinary art classes, I did. I just graduated that…So, I accomplished a lot.” (Client interview, Provider E, English).

Staff also believed that the program was impacting participants’ self-regard and pride in their identities, which contributed to feelings of self-efficacy around accomplishing goals. In the words of one staff member:

“Once they are here and they start participating and they start seeing how they are not alone, a lot of times just that cultural impact of like, you just be proud of being Latin. There’s a lot of really amazing things that just as a culture we have been able to achieve. So, you can be proud of that…you are part of this big community that everybody supports and all these different things. And you just start seeing that hope come up and that feeling of, ‘Yeah, I can do this.’” (Staff interview, Provider A).

To support effective service delivery, CARE included intensive, low-barrier outreach and engagement tailored to client needs and preferences. Providers averaged 2–3 contacts monthly for participants’ first 3 months enrolled, and 1–2 contacts monthly for the subsequent 10 months (Table 5). There was substantial variation in contacts across months and across clients. Some clients had months with zero contacts, while some had months with 10+ contacts. By the sixth month after enrollment, 53.1% of clients had zero logged contacts, indicating that they’d stopped engaging with the program.

**Table 5:** Contacts per active client and per month post-enrollment, by provider.

Category	Provider B		Provider C		Provider D		Provider E		All	
Total contacts	230		1,311		1926		2,576		6,043	
Contacts per active client										
Mean (std. dev)	7.4 (7.4)		15.1 (17.8)		38.5 (36.2)		10.7 (7.2)		14.8 (18.5)	
Minimum	1		1		2		1		1	
Median	5		9		26		9		9	
Maximum	29		112		154		40		154	
Month since enrollment	Active clients	Average contacts	Active clients	Average contacts	Active clients	Average contacts	Active clients	Average contacts	Active clients	Average contacts
0	31	0.6 (1.1)	87	3.0 (2.4)	50	3.8 (3.6)	240	2.2 (1.6)	408	2.5 (2.2)
1	31	1.4 (1.9)	83	2.2 (2.3)	48	4.6 (3.4)	240	2.4 (1.9)	402	2.5 (2.3)
2	31	1.3 (2.0)	79	1.5 (1.7)	43	4.4 (5.0)	223	2.0 (1.8)	376	2.1 (2.5)
3	28	0.8 (1.8)	73	1.7 (2.8)	41	4.0 (4.3)	194	1.8 (1.7)	336	2 (2.5)
4	27	0.6 (1.1)	68	1.4 (2.1)	38	3.4 (3.6)	159	1.5 (1.6)	292	1.6 (2.2)
5	26	0.6 (1.0)	63	1.3 (1.9)	37	3.3 (3.1)	116	1.4 (1.7)	242	1.6 (2.1)
6	26	0.3 (0.7)	61	1.1 (1.8)	35	3.1 (2.9)	91	1.0 (1.4)	213	1.3 (2)
7	25	0.7 (1.1)	58	1.2 (2.1)	31	3.9 (4.0)	61	0.7 (1.0)	175	1.4 (2.5)
8	19	0.4 (0.7)	53	1.3 (1.9)	26	4.4 (4.9)	36	0.8 (1.5)	134	1.6 (2.9)
9	14	0.6 (1.3)	50	1.2 (2.4)	23	5.1 (4.7)	27	0.6 (1.0)	114	1.7 (3.2)
10	9	0.2 (0.4)	46	1.3 (2.6)	15	6.4 (4.2)	19	0.7 (1.2)	89	2 (3.3)
11	9	0.6 (0.7)	41	0.9 (1.7)	13	4.8 (4.0)	12	0.8 (1.1)	75	1.5 (2.6)
12	9	0.2 (0.4)	36	0.9 (1.9)	11	3.2 (3.4)	10	1.2 (1.4)	66	1.2 (2.2)

Contact frequency also varied across programs. Provider B had fewer contacts and active participants than other programs, averaging under one contact monthly. Conversely, Provider D averaged 3–6 client contacts monthly over a full year of enrollment. This may be related to their higher-barrier screening process, which required interested incarcerated individuals to write a letter committing to recovery and frequent engagement with the program. For those willing to commit, Provider D offered particularly high-touch support, including meeting clients at release, bringing them clean clothes and comfort supplies, and transporting them home.

Provider staff emphasized relationship quality as much as interaction frequency, seeking to help clients feel seen and understood. Clients described how providers’ nonjudgmental, open-minded, and respectful approach made it feel safe to discuss drug use, criminal-legal history, or other sensitive topics. Clients appreciated program staff offering suggestions and support rather than directives, which fostered a sense of agency, confidence, and pride in accomplishments. Additionally, clients appreciated staff’s proactive outreach, willingness to support diverse needs, and consistent follow-through. One client described how outreach reflects staff’s caring:

“Well, what I really like is…they are on top of you. They worry about you. They’re always checking up on you, and if they even think you are slipping or if they feel you are on the wrong way…they’ll reel you in and try to talk to you, figure out what’s going on…They’re pretty good with that. They actually worry about you, and, like I said, for people like me…that’s big.” (Client interview, English, Provider D).

#### Community building and social support

In addition to individualized case management, CARE aimed to foster meaningful, supportive peer relationships among clients. Most providers cultivated these relationships by offering regular opportunities for clients to interact, relax, and learn together. Beyond weekly groups and drop-in hours, providers hosted group outings such as bowling, apple picking, fishing, and cookouts. Provider D started a basketball league and weekly barbershop nights. These activities helped clients engage, some for the first time, with local community resources. Additionally, some providers engaged current and former clients by inviting them to participate in advisory boards, volunteer at events, and share their reentry and recovery experiences with peers.

Supported by these activities, participants described a feeling of “brotherhood” at the programs — to many, the community felt “like a family.” When effective, this community helped clients share, learn, and support each other. However, physical space and program policies sometimes impacted engagement quality. Provider A’s clients described how the policies of the co-located housing program inhibited their agency around group engagement: they were mandated to attend daily groups or risk housing loss, and had limited privacy. Provider C2 offered limited in-office community-building opportunities because of space constraints, and the conference rooms used for groups were not conducive to client comfort. Finally, Provider D excluded people who were using drugs from some group activities “for the sake of other participants,” limiting their access to the community-building components of the program.

#### Facilitating access to evidence-based treatment and harm reduction services

All interviewed clients felt they could have open and honest conversations about substance use with staff, with several specifying that this felt safe because many staff had personal experience with addiction. Across providers, staff aimed to be nonjudgmental when discussing substance use and acknowledged the importance of harm reduction. Yet, specific approaches to supporting SUD treatment and harm reduction varied according to organizational infrastructure, experience, individual perspectives on abstinence, and client characteristics. Providers C and E had the most robust approaches, with staff consistently emphasizing safety and connecting clients to resources tailored to their current needs, including naloxone, safer use supplies, fentanyl testing, and medications for opioid use disorder (MOUD), among other services. Both organizations had SUD service infrastructure and experience pre-dating the grant; Provider E had an in-house center offering some harm reduction services, and as FQHCs, Provider C and its subcontractor had in-house capacity for MOUD and harm reduction. Both providers also had community partners for additional treatment and harm reduction services. A staff member described their approach as follows:

“I cannot stop a person from doing what they want to do or using what they want to use…[Addiction is] a medical condition…So I have to give them all the tools. I can give them the counseling, I can [help them get] treatment…I can give them information on where they can get clean needles, clean supplies, clean pipes. “Do not share your needles. Get tested for HIV, Hep C.”…When they feel comfortable and they feel that you are sincere on the information that you are giving them and the help that you are giving them, they tend to open up to you.” (Staff interview, Provider C1).

While Provider A had in-house MOUD infrastructure and offered harm reduction education, naloxone, and community resource information, their ability to support safety for people using substances was limited because many CARE clients lived in their abstinence-required residential treatment or sober housing facilities. As a staff member said, “There is a strong structure here to make sure that they are not using.” Consequently, clients perceived that abstinence was required for CARE participation, with one saying, “They do not [provide safer use resources]. You cannot take drugs here” (Provider A, Spanish). Similarly, Provider D was perceived as abstinence-oriented, despite offering naloxone, fentanyl test strips and some harm reduction education. One client said, “I really have not had that conversation [about safer use]. They’d rather us not use drugs. Yeah. I had more conversations about how important it is to be sober…But they do teach us about Narcan…I always got Narcan in my bag” (Provider D, English).

Provider B entered the grant with limited experience and infrastructure for evidence-based SUD services, and prioritized people with alcohol use disorder for most of the CARE pilot rather than those at high risk of overdose. Qualitative data indicated that staff conceptualized the program as primarily focusing on community reintegration rather than overdose prevention, and that some organizational leadership initially avoided recruiting people who use opioids, perceiving them as less likely to succeed. Consequently, Provider B did not emphasize access to MOUD or overdose prevention services for much of the pilot period. In year three, they hired a staff member with prior experience working with people who use drugs, and sought training on evidence-based overdose prevention. They began providing fentanyl testing strips, stocking naloxone, and more actively supporting clients who were using MOUD. Staff were open to harm reduction and interested in training on how to work with people who use high-risk substances, but they lacked confidence in implementing these practices.

Almost all participants and staff believed the program had prevented overdose deaths through one or more of the following mechanisms: providing harm reduction information and supplies; inspiring participants to “do the right thing” and maintain sobriety; being a safe and supportive partner through return-to-use; providing housing and counseling; and keeping participants “busy” through education, employment, and group activities. Provider B was a notable exception: staff expressed that the substance use histories of the people they were recruiting were such that they anticipated no meaningful impact on overdose.

## Discussion

The CARE program model featured practices affirmed by the literature to support improved service engagement and outcomes (see Table 2 for additional information and references). The evaluation assessed the implementation of these practices in a multi-site, grant-funded program aimed at people at acute risk of overdose, and identified key facilitators and challenges. With the exception of pre-release HOC engagement, which relied on factors not fully under providers’ control, the core features of the CARE model were feasible for most providers to implement. Participants expressed appreciation for these features, and associated them with positive outcomes in their own lives. However, due to some providers’ recruitment practices, these services were not all successfully targeted at individuals at highest risk of overdose. Additionally, reach and case management implementation were uneven across providers. For example, total enrollments ranged from 71–316, the pre-release recruitment proportion from 10.1–47.9%, the proportion of clients with history of opioid misuse from 17.9–75.8%, ISP completion from 31.1–87.9%, and mean contact frequency from 7.4–38.5 per active client. Qualitative data provided insights into implementation practices underlying quantitative trends and illuminated recruitment approaches and challenges, organizational characteristics influencing service delivery, and programmatic variations across providers. Overall, success depended to a large extent on providers’ existing capacity and infrastructure, a finding consistent with implementation science frameworks (17). The most successful organizations in this study tended to be large, long-established, experienced with implementing grant-funded programming, and experienced delivering evidence-based SUD and harm reduction services.

There can be tension between the desire for programs to be run by emerging, community-led organizations, and the difficulty of implementing complex models like CARE that require experience and expertise in a number of areas. Agencies should consider partnership or coalition-based approaches that bridge this gap (18, 19). Given implementation complexities, funding structures should incorporate organizational readiness considerations, offering additional support and training for early-stage organizations, and a pilot period with clear off-ramps. Additional considerations for physical infrastructure, staffing, cultural responsiveness, collaboration with carceral facilities, and SUD treatment approaches are detailed below.

### Physical infrastructure

While some providers used grant funding to carve out dedicated physical space for delivering new services, others used it to support existing service delivery or invest in shared spaces. This had implications for recruitment and service delivery. For Provider A, co-location with sober housing programs made it difficult to implement the program in a truly low-barrier way that meets people wherever they are in recovery, affirming a known challenge with abstinence-based housing (20). The providers that created dedicated physical space (C1 and E) had the most implementation success, though they were also larger and more experienced in grant administration and SUD support.

### Staffing

Hiring staff with lived experiences of SUD or incarceration was essential for participant engagement, and justified the administrative burden it created in some counties. Staff with shared identities, languages, and neighborhood backgrounds were also viewed positively. However, recruitment and retention was challenging due to demanding work, high candidate expectations, and compensation constraints, and turnover disrupted the relationships that drove client engagement. Organizations implementing these programs must build supportive working environments, and compensate staff appropriately for their contributions and expertise (21–25).

### Cultural responsiveness

All funded providers, regardless of their size and experience, leveraged expertise in their own communities to facilitate participant engagement, and this was received as respectful, authentic, and trustworthy. However, combining two distinct populations—Black and Latino men—into one program resulted in the underrepresentation of Black men in the participant pool. Although these populations face overlapping barriers to accessing SUD care, including stigma and economic exclusion (26–28), distinct cultural populations deserve distinct approaches and resource allocation in proportion to overdose rates (29).

### Pre-release recruitment

Providers’ relationships with local HOCs determined their ability to recruit and engage incarcerated people before release. This observation is aligned with evidence that the characteristics of a carceral facility may influence the service utilization of people imprisoned there (30). Though some providers marginally improved their relationships with the HOCs, others failed to gain access during the pilot period. In considering feasibility of pre-release engagement, programs should carefully consider current and prospective access, and prepare alternative approaches for reaching the focus population.

It is also important to note that program recruitment began during the first years of the COVID-19 pandemic. Rapidly shifting and variable policies enacted in response to the pandemic by the discrete county Sheriff’s Departments included widespread suspension of in-person visitation and rehabilitative programming (31, 32). These approaches plausibly impacted the program’s recruitment of and service provision to incarcerated people during the pilot period.

### Evidence-based SUD recovery and harm reduction supports

Organizations experienced in evidence-based SUD treatment and harm reduction services delivered those services in culturally-responsive, person-centered ways. However, the smaller, less experienced agencies expressed stigmatizing views of drug use, screened out people at high risk of overdose, and/or excluded people who currently use drugs or relapsing participants from groups and community activities. These practices are at odds with overdose reduction goals (33).

All communities deserve equitable access to services and accurate, non-stigmatizing information about treatment and support options so that each client can choose their own path. While people under parole or probation face the threat of legal revocation or jail time for relapse, this risk should not preclude individual agency. Practitioners should address these potential harms while facilitating access to care and supporting people to make informed choices about their own safety and recovery (34). Overall, funders and programs should monitor implementation for harmful narratives and approaches, and pursue technical assistance and training to counter them when necessary. Further, overdose prevention must be explicitly prioritized in order to prevent programs from diverting limited resources from those at highest risk.

### Future directions for outcome measurement

Data from this implementation evaluation suggest future directions for rigorous outcome measurement. Future evaluations should examine the impact of CARE on overdose deaths, as this was the intended long-term outcome underlying the program design. In addition, ISP data and client feedback about social support highlight the importance of taking a holistic view of recovery outcomes. Metrics of success should include not only traditional outcomes related to drug use and SUD treatment, but also measures of overall psychological wellbeing, social support and social connection, housing and financial stability, progress toward employment, and overdose risk. Finally, future evaluations should assess how program engagement and outcomes are affected by socioeconomic factors such as clients’ educational attainment, housing status, and employment.

## Data Availability

The datasets presented in this article are not readily available because consent procedures for qualitative data do not allow for data sharing, and data from state databases are not approved for sharing outside of designated study team members. Requests to access the datasets should be directed to Ranjani Paradise; rparadise@icommunityhealth.org.

## Footnotes

1 Program name has been changed for confidentiality.
